# From values to choices: the mediating role of career adaptability of university engineering students

**DOI:** 10.3389/fpsyg.2025.1712488

**Published:** 2025-12-16

**Authors:** Dan Wang, Bohan Liu, Yue Liang, Di Wu, Yujie Duan, Dan Zhang, Chao Song

**Affiliations:** 1 College of Education Science, Jilin Normal University, Siping, Jilin, China; 2 Institute of Education, University College London, London, United Kingdom; 3 School of Psychology, Nanjing Normal University, Nanjing, Jiangsu, China

**Keywords:** career values, career choice, career adaptability, university students, social cognitive career theory

## Abstract

**Introduction:**

Career choice is a complex and consequential process for university students, and it shapes their long-term career trajectories. This study examines the impact of career values on university engineering students' career choices and investigates the mediating role of career adaptability in this process.

**Methods:**

Through semi-structured qualitative interviews with eleven university students, the findings identify two clusters of career values: intrinsic career readiness (e.g., foundational mindsets, professional knowledge as a career pillar), and extrinsic career aspirations (e.g., pursuing leadership, career achievement).

**Results:**

These values map onto four key career choice considerations: personal growth opportunities, identity construction, work compensation, and life pursuit. Career adaptability, comprising concern, control, curiosity, and confidence, emerged as a psychological mechanism that enables students to translate career values into informed and strategic career decisions. It helps them manage career uncertainties, pursue self-directed goals, and align their choices with both personal meaning and external demands.

**Discussion:**

By highlighting this value–adaptability–choice pathway, this study contributes to career construction theory and extends the social cognitive career theory (SCCT), particularly in the context of collectivist cultures. It also offers practical insights for educators and career advisors aiming to design interventions that foster value clarification and adaptability development among undergraduate engineering students.

## 1 Introduction

Career values refer to the importance individuals attach to different aspects of work (Rokeach, 1973), such as job outcomes and personal fulfillment (Hirschi, 2010). As stable psychological tendencies, they provide lasting motivational guidance for career choice and job satisfaction (Sortheix et al., 2015).

University students' career values vary across disciplines. Humanities students often prioritize intrinsic motivations and social impact (Byabashaija and Katono, 2011), while business students focus on financial success and professional growth (Kidwell, 2001). Engineering students, however, study and transition into labor-market contexts that are distinct and warrant closer attention (Sheppard et al., 2009). Recent work on university students' career values remains concentrated outside engineering, particularly in arts, humanities and business-management contexts, where scholars continue to examine value-related decision-making, employability orientations and perceived outcomes (e.g., first-year Arts/English/Language undergraduates' decision processes; humanities employability or values debates) (e.g., Lennox, 2025; Robson et al., 2023). By contrast, engineering-specific investigations remain comparatively few. New studies have characterized engineering university students' agentic vs. communal career values and explored choice or retention mechanisms and career attainment in engineering programs, yet this is a developing strand rather than a saturated literature (Dori et al., 2024; Lee et al., 2024). This pattern suggests that university engineering students' distinct value orientations and decision processes may not be fully captured by findings derived from non-engineering cohorts, highlighting the need for discipline-specific analyses.

While career values shape career preference, career adaptability, influenced by career readiness, has been theorized as a mediating resource that links motives into adaptive responses (Savickas and Porfeli, 2012). Research has also reaffirmed the construct's contemporary relevance in higher education settings including Chinese student samples, through updated syntheses/validations and higher education applications (e.g., Demirtaş-Zorbaz et al., 2024; Hu et al., 2025; Soares and Taveira, 2024). Given the dynamic and competitive nature of engineering, this study examines how career adaptability mediates the relationship between career values and informed, strategic career choices.

By addressing these questions, this research provides a career adaptability-centered, qualitative account of how Chinese engineering university students' career values are translated into career choice considerations. Accordingly, we adopt a qualitative, mechanism-focused design to examine the value → adaptability → choice pathway, using the 4Cs of career adaptability (concern, control, curiosity, and confidence) as a sensitizing lens. The findings offer discipline-specific insights to support educators and career advisors in fostering effective planning and adaptive development.

### 1.1 Career values and career choices

Career values are critical motivators in job selection and career development (Balsamo et al., 2013). Hirschi (2010) links them to individuals' expectations about job characteristics and personal fulfillment, highlighting their role in shaping career choices and satisfaction. As stable psychological tendencies, career values provide a strong motivational basis that drives individuals to achieve desired end states (Rokeach, 1973) and guides how they evaluate career opportunities and pursue goals (Wang, 2021). They are also positively associated with related career resources and outcomes such as career adaptability, decision-making self-efficacy, and long-term career achievement (Taylor et al., 2014; Zhou et al., 2016). More recent evidence shows that personal values underlie young adults' career-related preferences during choice deliberation and predict downstream career outcomes, reinforcing their role as fundamental drivers of career behavior (Arthaud-Day et al., 2025; Lipshits-Braziler et al., 2025).

Career values are commonly classified into intrinsic and extrinsic dimensions (e.g., Hirschi, 2010). Intrinsic values relate to internal satisfaction and needs, such as autonomy, competence and self-fulfillment, whereas extrinsic values focus on material outcomes, including salary, benefits, and job security. From a self-determination perspective (Ryan and Deci, 2000), people with stronger intrinsic values are more likely to enjoy their job, persist in their pursuits, and better adapt to evolving environments (Sortheix et al., 2015).

These values often solidify during university, a period of identity formation and social role exploration. Emerging adulthood (ages 18–25) is a key phase for discovering values, constructing interests, life goals, and career orientations (Hunter et al., 2010). Through academic and social engagement, while gaining career-related experiences, students develop clearer expectations of what work should provide, such as personal growth, achievement, or stability (Jacobs et al., 2005). Consequently, career values directly inform students' preferences and help shape their early career decision frameworks.

However, the translation of values into action is not always straightforward and automatic. While career values guide overall preferences, they may not in themselves be sufficient for making effective or strategic career choices, especially under uncertainty or labor market constraints. This suggests that other mechanisms may be needed to facilitate the alignment between values and concrete decisions, motivating our focus on adaptability as the linking mechanism.

### 1.2 Career adaptability

Career adaptability refers to the ability to cope with unusual and uncertain problems in career development (Super and Knasel, 1981). As a multidimensional psychosocial construct, it comprises 4Cs—concern, control, curiosity, and confidence, which together empower individuals with psychological strength, flexibility, and resilience to effectively handle career transitions and challenges in career trajectories (Savickas, 1997). Building on this, Savickas and Porfeli (2012) identified career adaptability as a mediating resource: it is shaped by individual readiness and, in turn, influences adaptive responses, such as decision-making effectiveness and career exploration. For example, students with high adaptability are more capable of adjusting their mindsets during job search and demonstrate stronger links between their future work selves and job-search self-efficacy (Guan et al., 2014), thereby improving job-person fit and career decision quality (Koen et al., 2010). Career adaptability also predicts a range of positive career-related outcomes, including professional competence (Guan et al., 2016; Guo et al., 2014), job search success (Guan et al., 2014), salary (Guan et al., 2015), and career satisfaction (Chan and Mai, 2015). Recent scholarship has reaffirmed the conceptual clarity and cross-context robustness of career adaptability. Updated syntheses and short-form validations, including studies with Chinese student samples (e.g., Demirtaş-Zorbaz et al., 2024; Hu et al., 2025; Ma and Mohd Kosnin, 2024; Parola et al., 2025), support using it as an analytic lens in higher education. These findings reinforce the status of adaptability as a bridge connecting supports and orientations to enacted outcomes in university cohorts.

While substantial bodies of research have examined the direct effects of adaptability on career outcomes, its mediating role between deeper motivational constructs (e.g., career values) and enacted choices remains comparatively underexplored, particularly in qualitative mechanism accounts and in engineering higher education (Kumar et al., 2024; Oliveira and Marques, 2024). Addressing this gap, the present study centers adaptability as a dynamic mechanism through which career values are translated into strategic, realistic, and aligned decisions. Accordingly, this study adopts a mediating perspective, hypothesizing that career values, though foundational, influence career choices through adaptability.

## 2 Theoretical basis

Bandura's (1986) reciprocal determinism suggests how individual behaviors arise through interactions between internal thoughts, emotions, and the surrounding environment. Building on this foundation, Social Cognitive Career Theory (SCCT) (Lent et al., 1994) has provided a robust framework for examining how individual traits, environmental factors, and adaptive behaviors collectively influence career development. It highlights the dynamic interplay between cognitive and adaptive processes in guiding career decisions: career values may be conceptualized as antecedents that shape career adaptability, which in turn influences how individuals make career choices. For instance, for engineering students, values such as innovation, technical expertise, and professional growth may play a formative role in determining their adaptability and subsequent career decisions. However, despite subsequent SCCT extensions, such as the social cognitive model of career self-management (Lent and Brown, 2013), career values remain overlooked, even though they are fundamental drivers of career-related behaviors and decision-making (Hirschi, 2010; Wang, 2021).

In parallel, adaptive career behaviors are acknowledged within SCCT as crucial resources (Lent and Brown, 2013). Career adaptability, conceptualized as the 4Cs (concern, control, curiosity, confidence), is treated as a psychosocial resource that helps individuals manage vocational tasks, transitions, and traumas (Savickas and Porfeli, 2012). Contemporary higher education studies continue to employ adaptability as a proximal resource associated with students' career certainty, academic well-being and life satisfaction, and with readiness for the school-to-work transition (e.g., Oliveira and Marques, 2024). This positioning aligns with contemporary higher education models that integrate career construction and socio-cognitive traditions, placing adaptability as a proximal mechanism that connects supports and orientations to students' academic and career outcomes (Soares and Taveira, 2024).

This study integrates career values into SCCT, conceptualizing them as motivational antecedents that influence career decisions through the mediating role of career adaptability. The 4Cs of career adaptability are used as a sensitizing lens, not an *a priori* coding frame, to interpret how inductively derived mechanisms connect values to decision processes. By clarifying this pathway, this study aims to extend SCCT in higher education and offer a more comprehensive understanding of how engineering university students' value-driven motivations interact with adaptive behaviors to shape career choices.

## 3 Methods and materials

This study employs semi-structured qualitative interviews to explore how university engineering students' career values inform their career decisions, as revealed through their personal reflections on daily activities, academic journeys, and descriptions of their future jobs. The open-ended interview design encouraged participants to articulate their perspectives freely, yielding rich, contextual data grounded in authentic experiences.

### 3.1 Setting and participants

We recruited eleven engineering students from universities in a third-tier city in China using a convenience sampling method. To ensure variation in perspectives, participants were purposefully selected to represent different genders, grade levels, student governance involvement, and engineering sub-disciplines (Electronic Information Engineering, Optoelectronics, and Communication Engineering).

This heterogeneity enabled the exploration of how different student profiles perceive and prioritize their career values, as well as express their career-choice considerations. Participant details are provided in Table 1.

**Table 1:** Participants' information.

**ID**	**Gender**	**Grade level**	**Student officer^a^**	**Course**
A	Female	Year 1	Yes	Electronic information engineering
B	Male	Year 3	Yes	Electronic information engineering
C	Male	Year 3	No	Optoelectronics
D	Male	Year 3	No	Communication engineering
E	Female	Year 2	No	Optoelectronics
F	Male	Year 3	Yes	Communication engineering
G	Male	Year 2	Yes	Electronic information engineering
H	Female	Year 1	Yes	Communication engineering
I	Male	Year 3	No	Electronic information engineering
J	Female	Year 2	Yes	Optoelectronics
K	Male	Year 1	No	Communication engineering

^a^In China, student officer role often implies students have specific duties or formal positions within student governance and organizations, such as those on a student council or in other representative roles.

### 3.2 Data collection

An interview protocol was developed to align with the research objectives, focusing on eliciting participants' career values and career-choice considerations. The structure and wording were refined through expert consultation and pilot interviews with four non-participant university students. Insights from this pre-testing informed the final protocol used in the main study.

All participants provided informed consent for audio recording. Each interview lasted 30 to 63 minutes, generating 11 valid transcripts. To ensure confidentiality, all identifiers were removed during transcription. Verbatim transcriptions were carefully cross-referenced against audio recordings for accuracy. The final dataset comprised approximately 100000 words, which researchers repeatedly and thoroughly reviewed to build familiarity ahead of data analysis.

#### 3.2.1 Interview protocol design

The semi-structured interview targeted three linked domains central to the research focus: (a) career values, (b) career direction and concrete choice factors, and (c) translation from values to choices. A funnel sequence (broad → specific) was used, with neutral probes to avoid leading.

1) *Career value judgements (what matters and why)*.
  Core prompts included: “What does an ideal career look like to you?”; “What principles do you use to judge a ‘good job'?”
  Typical probes: “Could you give a recent example?”; “How did you form that view?”
2) *Career direction and choice factors (what you seek and how you trade off)*.
  Core prompts included: “What roles or sectors attract you most, and why?”; “Which factors matter most when choosing a job (e.g., growth, identity fit, remuneration, stability, location/lifestyle)?”; “How do you weigh these factors when they conflict?”
  Typical probes: “Tell me about a time you choose between two options.”
3) *From values to choice (mechanism and alignment)*.
  Core prompts included: “How have the values and factors you mentioned influenced a specific decision you made (or are planning)?”; “Looking back, does the decision align with your original values and aspirations?”
  Typical probes: “What made alignment easier or harder (information, constraints, support)?”

#### 3.2.2 Generic probes aligned with adaptability domains (not foregrounded during interviews)

Although the protocol did not foreground “career adaptability”, the interviews included neutral prompts on planning horizons, agency in setbacks, exploratory actions and confidence, which later provided material to examine adaptability-related processes. For example:

1) “How far ahead do you plan?” (future orientation)
2) “When plans change, what do you do to stay on track?” (agency/ownership)
3) “What actions have you taken to explore options?” (exploration)
4) “How confident do you feel about executing this plan, and why?” (self-efficacy)

These prompts were phrased generically and were not presented to participants as measuring adaptability. Rather, they served as sensitizing concepts to ensure coverage of decision-making processes without imposing *a priori* categories. All participants received the same guide, and minor clarifying follow-ups were permitted to pursue emergent mechanisms.

### 3.3 Data analysis

Thematic analysis, grounded in interpretive phenomenology, was used to identify recurring themes and sub-themes across the interview data and to explore participants' lived experiences in depth. To enhance rigor and consistency, three researchers worked collaboratively. Two researchers independently coded an initial subset of transcripts to calibrate code meanings; discrepancies were resolved through three-way discussion, and the codebook was refined. The third researcher also acted as a methodological auditor, reviewing coding decisions and memos at milestones and challenging category boundaries where needed. The remaining transcripts were then coded with the stabilized template, with periodic calibration meetings.

The primary inductive analysis proceeded in three steps:

1) Familiarization: immersing in the data, reading transcripts repeatedly, and generating initial codes to capture key ideas;
2) Theme development: iteratively reviewing the codes and grouping them into broader themes that reflected shared experiences and insights, emphasizing recurring patterns across the interviews;
3) Refinement and structuring: analyzing relationships between themes, integrating related themes into overarching categories that characterize career values and career choice patterns.

This structured approach provided a robust foundation for understanding how career values are expressed and operationalized in students' career-related decision-making.

In a subsequent, theory-informed sense-making phase, we used career adaptability (4Cs) as a sensitizing lens to interpret how the inductive themes connect to decision processes (e.g., future orientation/Concern, agentic regulation/Control, exploratory action/Curiosity, confidence talk/Confidence). The lens served to clarify rather than pre-empt participants' meaning-making and is used to articulate mechanism narratives (values → adaptability processes → choices) and to relate the findings to the wider literature. Earlier transcripts were revisited where the lens added explanatory value.

### 3.4 Ethics approval statement

Ethical approval for this study was obtained from the Science and Technology Ethics Committee of Jilin Normal University (approval number: KJLL20250404). Written informed consent was obtained from all participants prior to data collection, including consent for participation and for the publication, and all interviews were conducted in a confidential setting.

## 4 Findings

While multiple themes were identified, these are organized into two overarching analytical domains from the interview data:

1) Career values
2) Career choice considerations

Within each domain, specific themes and sub-themes are illustrated through excerpts and interpretation.

### 4.1 Career values of engineering students

The participants' career values, focus on early career readiness and long-term developmental aspirations.

#### 4.1.1 Career readiness

##### 4.1.1.1 Starting pragmatic and foundational mindsets

Participants viewed entry-level positions as necessary stepping-stones for skill development and long-term career success, reflecting workplace norms and wider societal expectations for new graduates in China. Fresh graduates usually “*cannot find a high-paying job at the beginning, so you must start from the entry level to earn promotions*” (Respondent A), and they must “stay grounded, take each step seriously” (Respondent I). This pragmatic orientation is shaped by competitive job markets, limited opportunities, and a cultural emphasis on incremental success.

Several participants viewed entry-level positions in reputable companies as valuable opportunities to observe organizational operations and identify potential growth paths; others linked the mindset to student-era extracurricular roles that fostered resilience and upward mobility (Excerpts A1–A2 in Supplementary Materials Section-A). These reflect how pragmatic and foundational values are formed and sustained across different stages of participants' educational and professional trajectories.

Respondent F stressed humility, diligence, and proactive learning: “*Less talk and more work is the best policy. Be practical, enrich yourself, ask for advice from more experienced staff, be sincere, and help others*.” Such concrete actions, including seeking mentorship and building interpersonal goodwill, turn early-career professionals into foundations for long-term advancement.

Together, these perspectives treat initial roles and challenges not as obstacles but as essential steps toward durable development. Foundational work and sustained effort cultivate perseverance, repeatedly emphasized as vital in navigating early-career setbacks and adapting to professional environments. This emphasis on practicality, persistence, humility, and proactive learning explains why participants embraced entry-level positions as growth opportunities, rather than compromises.

##### 4.1.1.2 Cultivating independence and self-driven success

Many participants, especially those from rural or underprivileged backgrounds, saw self-reliance and personal effort as core values. Rather than relying on external supports, they placed trust in their own initiative.

“*The only way to is to work hard and study hard. Like me, I don't have money, power, or influence, so I can only rely on my own efforts*.” (Respondent H)

This statement reflects more than socioeconomic constraint: a principled belief in self-driven success and a refusal to depend on privilege, and an embrace of personal responsibility in navigating career paths.

“*My family is from a rural area, my parents farm. I've worked with my own hands and seen how hard life is… I have to study and find a good job by my own efforts*.” (Respondent E)

Here conviction turns into concrete action. Studying hard, working independently, and striving for opportunities are not simply means to an end, but markers of worth and resilience, showing how students without structural advantages derive confidence from disciplined effort. Several also acknowledged the difficulty of staying motivated, noting moments of fatigue that demanded renewed determination (Excerpt A3 in Supplementary Materials Section-A).

Together, these accounts articulate a value of self-reliance and self-driven success. For these participants, success lies more in the process than in the outcomes, with persistent effort and inner strength being key to claiming agency over the future. Independence and perseverance thus form the moral fabric of their career identities, providing the motivation and psychological resources (control, resilience, and confidence) necessary to navigate early career choices under uncertainty.

##### 4.1.1.3 Valuing technical mastery and adaptive skills

Participants consistently emphasized the importance of developing versatile career competencies that combine domain-specific expertise, emotional intelligence (EQ), and strategic problem-solving. These are not only for employability, but as principles guiding long-term growth.

“*If you want to stand out, you must have certain domain-specific abilities*… *Of course EQ is important in this environment, so that you can use your strengths and abilities more rationally*.” (Excerpt A4 in Supplementary Materials Section-A)

Here, technical mastery was essential not only for task performance but also for earning trust and visibility. Emotional intelligence (EQ) complemented this by helping manage workplace dynamics and maximizing personal strength through interpersonal effectiveness. This dual focus reveals a value orientation rooted in self-improvement and strategic collaboration.

“[*I]mprove your ability to work and solve problems. You must be able to find the entry point quickly*… *Find a method that suits you*… *so that when you encounter a problem, you can identify and implement a solution more efficiently*.” (Excerpt A5 in Supplementary Materials Section-A)

This reflection highlights a mindset focused on adaptive problem-solving, personalizing strategies, learning from experience, and acting decisively. Respondent G's account illustrates how problem-solving evolves from a discrete skill into a critical mindset for managing workplace complexity. Rather than reacting passively, participants applied strategic thinking and refined their methods through real-world practice.

These perspectives reflect a shared value that competency arises from continual refinement of technical knowledge through adaptive application. Mastery and strategic problem-solving were seen as intertwined tools for building trust, credibility, and managing complexity. Competency was actively constructed through practice, reflection, and responsiveness to context, which positions participants as growth-oriented, resilient professionals.

##### 4.1.1.4 Valuing professional knowledge as career pillar

Participants recognized professional knowledge not simply as technical tools, but as a pillar of long-term development. Rather than focusing on immediate, task-specific skills, they valued the deep disciplinary grounding and conceptual clarity offered by university education, which fostered confidence in tackling future demands and unfamiliar challenges.

“… *[E]nhancing professional knowledge through internship practice, like needing to design a circuit, laying out the actual thing, so that the profession can be enhanced by solving practical problems to really learn in*.” (Excerpt A6 in Supplementary Materials Section-A)

This perspective reflects the synergy between academic theory learning, hands-on experience, and real-world application. For students like D, internships were not solely résumé-builders but key moments to align subject knowledge with practice and prepare for real workplace demands.

Participants also appreciated academic platforms as critical career resources. Despite acknowledging curriculum-industry gaps, they valued universities as launching pads for lifelong learning and cognitive adaptability:

“*The school provides a professional platform, with the ability to learn professionally, it is easy to learn new things even if you need to go to the workplace*.” (Respondent I)

Professional knowledge here is seen as transferable capital that prepares students for known and unforeseen challenges. Academic training supports their capacity to learn, interpret, and adapt in evolving environments.

In this light, professional knowledge becomes a pillar for career navigation and a durable asset, as Respondent I put it: the “fundamental and hardware for job search”, built through both formal instruction and peer learning. This demonstrates a value grounded in continuity, preparation, and strategic foresight, positioning knowledge as substance and investment for future adaptability.

#### 4.1.2 Career aspirations

##### 4.1.2.1 Pursuing leadership for autonomy and influence

Most participants strongly aspired to leadership positions, expressing a desire to “be the boss”, become a “strong woman”, or “start a business”. These ambitions reflect a core career value: striving for autonomy and influence. Rather than remaining in purely technical roles, students emphasized developing leadership capabilities to shape their career trajectories and expand their impact. For instance:

“*I want to become a boss, not just a technician. I want to make the products I want and have my own business*.” (Respondent B)

This response encapsulates a desire for autonomy through entrepreneurship, positioning business ownership as both self-fulfillment and career agency. Participants view entrepreneurship as an extension of their technical foundation, requiring practical experience and strategic vision to transition from execution to direction.

Others saw senior management as a platform for personal and professional growth:

“*My career plan is to enter senior management, where I will meet better people in a better environment*.” (Respondent D)

This highlights a career value centered on leadership as a gateway to elevated opportunities: gaining access to influential networks, advancing personal competencies, and contributing at a broader level. For these participants, beyond being a status symbol, leadership is also a mechanism for long-term adaptability, vision-building, and self-directed career evolution.

##### 4.1.2.2 Challenge-driven motivation and growth

Participants described challenges as turning points and motivators in career growth. Rather than perceiving limitations as discouraging, they viewed them as catalysts for self-reflection and goal realignment. These included from technical skill gaps, social interaction difficulties, and comparison-induced anxiety. For instance:

“*[M]y seniors were very capable, but there were still things they weren't satisfied with during their job search. So I can't help worrying, if I am not as strong as them, what will it be like for me?*” (Respondent E)

“*During my internship, I encountered various technical issues, as well as challenges related to interpersonal interactions in society*.” (Respondent H)

Such experiences often triggered a reassessment of career direction, showing how discomfort became a driver of self-improvement. Instead of avoiding these shortcomings, participants worked to close gaps and refine goals. Respondent H then described learning from senior peers translated challenges into planning:

“*The challenges allow me to consciously learn skills required by the company, then preparing myself for real-world requirements… I gained insights into the skills needed for specific professions and used this to prepare myself with relevant goals, such as becoming a hardware engineer*.” (Excerpt A7 in Supplementary Materials Section-A)

Similar insights appeared elsewhere. Some described how tutoring discomfort built communication skills; others stressed continuous learning to keep pace with industry shifts. These reflections further demonstrate how participants embraced everyday pressures as drivers of growth (Excerpts A8–A10 in Supplementary Materials Section-A).

Ultimately, participants treated adversity not as a barrier but as training for adaptability. By transforming uncertainty into action, they strengthened career focus, psychological readiness, and the capacity to navigate future demands. This career value that embraces challenge as developmental fuel underpinned long-term adaptability, equipping students to learn, pivot, and thrive in evolving work environments.

##### 4.1.2.3 Recognition and self-directed accomplishment

Participants expressed strong value placed on career achievement, tied to both external recognition and personal fulfillment. Their aspirations reflected not only the symbolic status of professional roles but also a desire for prestigious, self-authored success.

The appeal of “white-collar” work lay in its prestige and dignity. These roles symbolized social recognition and personal worth:

“*I imagine enjoying the white-collar lifestyle… [H]aving my own workspace… meeting in a proper room. That makes me feel accomplished… I'd have more freedom to carry out tasks in my own way, shape my own little world at work*.” (Excerpt A11 in Supplementary Materials Section-A)

Alongside “being recognized by leaders”, this illustrates how clear routines and organizational formality offer prestige and a platform for autonomy, efficiency, and professional identity. Their pursuit of achievement is thus shaped by a dual desire: to be respected in structured environments and to exercise agency within them.

Others described success through ownership and independence. Building one's own industry was deeply personal rather than a technical ambition:

“*I always had this expectation to make the products by myself, to have my own business*… *that gives me a sense of achievement*.” (Respondent F)

This highlights a vision grounded in initiative and self-direction, where drive and fulfillment come from creating something of one's own.

Whether seeking respect through formal roles or building success independently, they valued autonomy, initiative, and competence. Recognition from leaders, clearly defined roles, and societal approval reinforced their motivation. This dual emphasis reflects a future-oriented mindset, marked by agency, focus, and commitment as key features of adaptability.

##### 4.1.2.4 Aspiring toward satisfying work environments

Participants aspired to workplace environments aligned with their values, considering them essential for success and motivation. One participant emphasized fairness and organizational clarity in shaping willingness to commit:

“*The company should have clear and complete rules and regulations, with no unfairness. If you work more, you should be paid more. I also care about future security, and whether the company offers space for growth and upward mobility. If so, I'll be motivated to work hard*.” (Respondent J)

This highlights three priorities: fairness (reward for effort); structural clarity (rules and protections); and upward mobility (visible progression). For engineering students, these are practical motivators that fuel sustained commitment.

Respondent I added an emotional layer, describing how competitive energy and daily comfort shape their ideal workplace:

“… *[A] competitive atmosphere*… *motivates me to work harder and… a comfortable environment*… *makes me more inclined to enjoy the work*.” (Respondent I)

Here, healthy competition stimulate improvement, while emotional comfort underpins well-being, underscoring a dual need for challenge and support.

Other participants described additional environmental features. Some stressed learning opportunities and variety, others sought reputable or growth-oriented firms; and some highlighted stability and humanistic culture, especially in state-owned enterprises, where long-term contributions are respected (Excerpts A12–A14 in Supplementary Materials Section-A). These preferences reflect a desire for settings that are secure yet dynamic, meaningful yet socially endorsed.

Together, engineering students' career aspirations extend beyond personal success to include the environments they aspire to thrive in. Their ideal workplaces combine fairness, development, stability, and recognition, revealing an adaptive, future-focused mindset attentive to both internal growth and external fit.

#### 4.1.3 Summary of the engineering university students' career values

Interview data revealed two central dimensions of participants' career values: readiness and aspiration.

*Career readiness* reflects various practical entry strategies, including pragmatism, independence, skill development, and valuing professional knowledge, highlighting career preparation and adaptability. *Career aspirations* encompass personal ambition and workplace ideals, such as pursuing leadership, embracing challenges, achieving recognition, and seeking supportive environments. As early-stage professionals, participants were keenly aware of employment pressures and focused on gaining experience and refining competencies.

Table 2 summarized the full set of themes and sub-themes identified. These career values not only underpin participants' career visions but also inform the specific needs and tensions explored in the following section on career choice considerations (decision-making).

**Table 2:** Summary of engineering university students' career values: categories, themes, and sub-themes.

**Category**	**Themes**	**Sub-themes**
Intrinsic career readiness	Starting pragmatic and foundational mindsets	Recognizing entry-level work
		Hardworking and perseverance
		Appreciating practical and humble approaches
	Cultivating independence and self-driven success	Valuing self-reliance
		Achieving success through personal effort
		Building resilience through hard work
	Valuing technical mastery and adaptive skills	Striving for mastery and professional excellence through technical skills
		Applying EQ for strategic interpersonal functioning
		Embracing problem-solving and strategic thinking
	Valuing professional knowledge as career pillar	Commitment to continuous learning and practical growth
		Appreciation for academic platforms as critical resources
		Dedication to building specialist knowledge
Extrinsic career aspiration	Pursuing leadership for autonomy and influence	Valuing growth beyond technical expertise
	Embracing autonomy through entrepreneurship
	Aiming for senior management
	Challenge-driven motivation and growth	Commitment to self-awareness of shortages
		Proactive mindset for skill development
		Recognizing the role of external pressures in career clarity
	Recognition and self-directed accomplishment	Valuing recognition and professional identity
		Sense of career accomplishment
		Seeking structured environments to enable personal agency
	Aspiring toward satisfying work environments	Pursuing fairness and equal opportunities
		Prioritizing growth-focused work environments
		Valuing stability and human-centered workplaces

### 4.2 Career choice considerations of engineering university students

Building on the identified career values, this section analyzes the key considerations of these engineering students' career choices, organized under two overarching themes: value embodiment and materials needs. To maintain clarity and avoid redundancy, detailed quotations and interpretations are provided in the Supplementary Materials Section-B.

#### 4.2.1 Value embodiment: personal growth and identity construction

Participants' placed strong emphasis on choosing careers that fostered professional development. They sought environments offering external training programs, mentorship, and hands-on experiences to refine both technical and soft skills. Fair competition, supportive and collaborative teams, and growth-oriented cultures were particularly considered.

In parallel, participants prioritized career paths aligned with their personal attributes and work ethic, such as diligence, perseverance, commitment, positivity, and adaptability, and saw these as vital to sustaining motivation and continuous improvement. Careers were not just seen as jobs, but as platforms for expressing identity and pursuing meaningful, purpose-driven goals.

Identity construction further influenced their career choices. Many values roles where their expertise would be recognized and their contributions respected and acknowledged. Leadership recognition and the opportunity to influence others also emerged as important, reflecting students' aspiration for autonomy, impact, and social standing.

Together, these considerations reflect a drive toward self-improvement, professional identity, and meaningful contributions to society, indicating that university students seek careers aligned with who they are and who they wish to become.

#### 4.2.2 Material needs: practical considerations

In addition to value embodiment, material needs significantly influence engineering students' career choices. Two focal concerns were identified: work compensation and life pursuit, reflecting a strategic balance between financial security, career progression, and lifestyle satisfaction.

Work compensation includes salary and benefits, job security, and financial stability. Students often linked company size, reputation, and role complexity with better benefits and promotion prospects. These pragmatic views reflect awareness of competitive job markets and the desire for stability.

Lifestyle factors are closely tied to broader life aspirations. Students expressed a preference for workplaces near family, in livable cities, and with supportive organizational cultures. A positive working atmosphere and alignment with personal values were seen as essential for long-term job satisfaction. Some also prioritized affiliation with reputable institutions that enhanced their social identity.

In balancing compensation and lifestyle, students demonstrated a holistic approach that integrates practical needs with broader goals for personal and professional fulfillment.

#### 4.2.3 Summary of career choice considerations: diverse and multifaceted

Table 3 summarizes the dual considerations shaping engineering students' career decisions. Intrinsic dimensions consist of *personal growth opportunities* and *identity construction*, reflecting deep personal values. Extrinsic dimensions consist of *work compensation* and *life pursuit*, highlighting pragmatic concerns, including financial stability, job security, and overall lifestyle satisfaction.

**Table 3:** Results of career choice considerations of engineering university students.

**Category**	**Themes**	**Sub-themes**
Value embodiment	Personal growth opportunities	Professional skill development and growth
		Supportive and equitable growth environments
		Career development resources
		Alignment with personal attributes and work ethics
		Motivation and aspirations
	Identity construction	Professional respect
		Social recognition
		Self-fulfillment
		Leadership recognition
Material needs	Work compensation	Company size
		Company development prospects
		Salary
		Satisfying job description
	Life pursuit	Favorable geographical location
		Organizational reputation
		Working environment
		Distance from home
		Organizational cultural

The dual emphasis reveals a strategic mindset: these engineering students weigh both internal aspirations and external constraints to guide career planning. Their decisions reflect a well-rounded perspective that connects professional ambition with practical viability.

## 5 Discussion

### 5.1 The path connecting career values to decision-making actions

While the findings reveal the influence of career values on career decision-making, the mechanism behind this translation warrants further exploration. Drawing on Savickas' (1997) career adaptability, this study argues that they mediate the relationship between career values and career choices.

Engineering university students' career values encompass both *career readiness* and *aspirations*. Career adaptability provides a psychological framework that translates these values into choices (Savickas and Porfeli, 2012). The following sections detail each adaptability dimension and show how engineering students reconcile values with pragmatic concerns—value embodiment and material needs.

#### 5.1.1 Career concern

Career concern, the capacity to envision future possibilities and prepare for upcoming challenges (Savickas, 1997), is the first hinge linking values to career choice consideration, answering the question “*Do I have a future?*” Participants scanned horizons, compared alternative trajectories, and scaffolded concrete action plans (Excerpt C1 in Supplementary Materials Section-C).

Students framed decisions in terms of long-run pay-offs: early workplace experience vs. postgraduate credentials, rapid income accumulation vs. deeper expertise. Such forward thinking converts abstract values (e.g., self-reliance, professional mastery) into viable strategies (e.g., choosing an entry-level role that accelerates skill acquisition).

High concern fosters active career exploration and better employment outcomes (Koen et al., 2010) and energizes job-search behaviors (Guan et al., 2014; Kvasková et al., 2023). Recent higher education evidence similarly indicates that adaptability-related resources (e.g., concern) underpin career certainty and well-being during university (e.g., Soares and Taveira, 2024). Echoing these, the data suggest that by projecting students into plausible futures, concern mobilizes them to balance intrinsic goals with pragmatic constraints, activating the broader adaptability resources set and thus initiating the mechanism that translates articulated career values into informed, context-sensitive choices.

#### 5.1.2 Career control

Career control refers to the self-discipline and perseverance needed to steer one's own paths (Savickas, 1997), responding “*Who owns my future?*”. Participants described rejecting norms, setting self-defined goals, and re-calibrating plans. For example, a participant rejected the advice that “girls should seek stability”, instead targeting a private-sector role she had envisioned since childhood; another mapped a staged ascent to senior management, treating each entry-level task as deliberate skills accumulation (Excerpts C2–C4 in Supplementary Materials Section-C). Such narratives show how control translates values of self-reliance, recognition, and growth into sequenced, actionable strategies.

Control is associated with job-search intensity and better employment outcomes (Hirschi, 2010; Koen et al., 2010; Rujiwattanapong, 2024), and Chinese evidence shows its balancing role amid shifting labor demands (Zhao and Guo, 2010). Consistent with this, students here framed incremental progress—“start low, move upward”—as a hedge against market volatility. The sentiment parallels Xunzi's maxim: “Pile up little steps to travel a thousand *li*” (Schofer, 1993), underscoring perseverance as culturally resonant capital. This volitional resource also resonates with Fugate et al.'s (2004) employability framework, where self-discipline buffers market shocks.

Maintaining ownership while flexibly adjusting tactics enables students to align immediate choices with long-range aims. Career control thus solidifies the second link in the “values–adaptability–choice” chain, providing the volitional energy that sustains planned trajectories amid shifting constraints.

#### 5.1.3 Career curiosity

Career curiosity reflects exploration of self and opportunities (Savickas, 1997), addressing “*What do I want to do?*”.

Participants displayed an active information-seeking stance, scanning both macro labor trends and micro job specifics. At the macro-scan level, students emphasized tracking peers' outcomes to “understand the employment prospect of my major and use it as a reference basis for my career choice” (Excerpt C5 in Supplementary Materials Section-C). This allowed her to test whether industries promising rich professional-skill development and growth resources matched her mastery-oriented values. At the micro-probe level, Respondent E approached seniors “to find out current interview criteria and employer requirements” (Excerpt C6 in Supplementary Materials Section-C), gauging how well specific roles could deliver recognition, fair appraisal, and career advancement.

Such targeted exploration expanded their option set and sharpened market awareness. These mechanisms are empirically tied to superior decision quality and job outcomes through adaptability (Hirschi, 2010; Koen et al., 2010; Ran et al., 2023). By combining research with first-hand intelligence, curiosity enables students to reconcile intrinsic ambitions such as entrepreneurial autonomy, senior-management tracks with pragmatic filters such as company reputation, job description, and growth climate. This mirrors Taylor et al.'s (2014) and Pham et al.'s (2024) findings that curiosity spurs internships and targeted upskilling to verify person–job fit. It therefore locks in the third link of the “values–adaptability–choice” chain, grounding decisions in timely, context-rich evidence.

#### 5.1.4 Career confidence

Career confidence denotes the belief that one can master forthcoming career challenges (Savickas, 1997), answering “*Can I do it?*” and locks the final link in the “values–adaptability–choice” chain. Students who cherish technical mastery, self-reliance, and professional recognition leveraged that assurance when evaluating choice considerations such as high-skill roles, reputable employers, and leadership pathways. For instance, a participant proclaimed that strong professional knowledge would enable entry into top-tier firms and continued upskilling (Excerpt C7 in Supplementary Materials Section-C).

This self-efficacy converts inward values into outward ambition: targeting elite companies, accepting stretch assignments, and re-framing obstacles as learning opportunities. Empirical research shows confidence intensifies job-search effort and buffers labor-market shocks (Hirschi, 2010; Guan et al., 2014; Zheng et al., 2025), while providing the psychological resources that sustain proactive behavior during transitions (Alkal, 2025; Savickas and Porfeli, 2012). Chinese studies similarly indicate that confidence helps graduates balance rapid market change with long-term growth (Wang, 2021; Zhao and Guo, 2010).

By aligning mastery-driven values with audacious yet calculated choices, such as seeking firms known for rigorous development, clear promotion ladders, and industry prestige, career confidence enables students to reconcile intrinsic aspirations (excellence, recognition) with pragmatic filters (competitive salary, advancement prospects). It thus ensures resilience and strategic coherence when navigating volatile employment landscapes (Purohit et al., 2021).

*Mechanism summary*. The above analysis highlights how career adaptability acts as a psychological hinge linking career values to career choice considerations. Through its four dimensions, career adaptability enables engineering students to align intrinsic motivations with external constraints and reach informed, strategic career decisions. This mediating logic echoes value-driven decision frameworks (Rokeach, 1973) and recent Chinese evidence on industry-aligned planning (Yue and Zhou, 2016). Prior work shows that well-defined values feed directly into adaptability (Hirschi, 2010; Zhou et al., 2016; Guan et al., 2014). Overall, the qualitative account of this study also aligns with recent higher education evidence that positions career adaptability as a proximal resource predicting students' career certainty, academic well-being and life satisfaction, and bolstering readiness for the school-to-work transition (Oliveira and Marques, 2024; Soares and Taveira, 2024).

**Figure 1:**
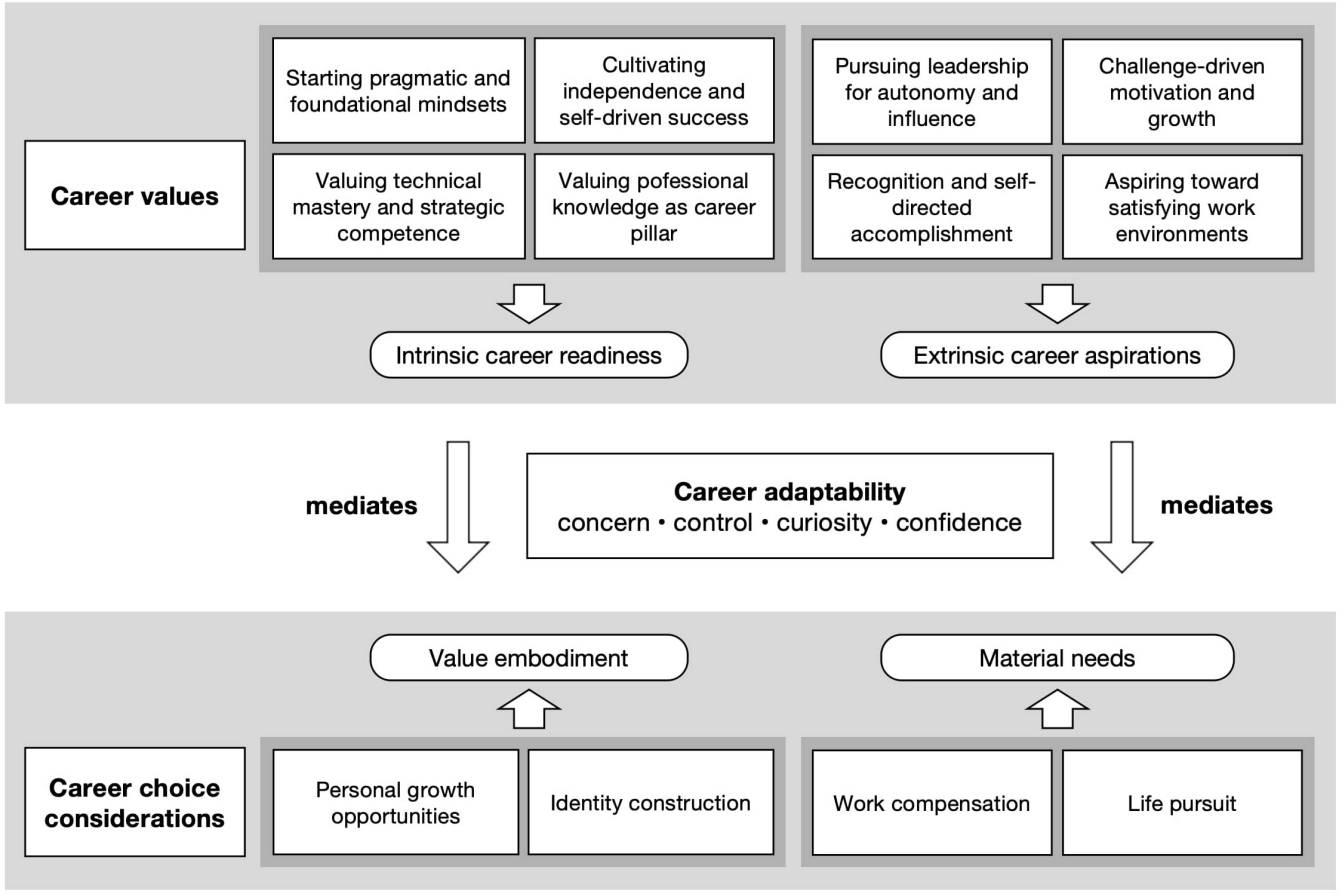
Mediating role of career adaptability linking career values to career choice among university engineering students.

Figure 1 illustrates how intrinsic career readiness and extrinsic career aspirations, via adaptability, translate into value embodiment and material needs considerations.

### 5.2 From values to career choices: four observed decision pathways

Participants' career aspirations ranging from software engineering to entrepreneurship, civil service and teaching show how clear values, reinforced by adaptability, steer choices that balance self-fulfillment with financial security and job stability. This pattern confirms that well-defined values motivate action, while adaptability helps manage uncertainty (Hirschi, 2010; Lipshits-Braziler et al., 2025; Zhou et al., 2016).

Engineering students favor jobs that offer *personal development opportunities*, such as internships, technical training, and leadership tracks. Adaptability motivates them to seize such growth options that align with their intrinsic values (Pham et al., 2024; Taylor et al., 2014; Zhou et al., 2016). They also weigh *identity construction*, using adaptability to align values like self-worth and leadership aspirations with roles that validate their professional identity (Arthaud-Day et al., 2025; Balsamo et al., 2013). *Work compensation* is another central concern. Students assess salary, benefits, and employer reputation in light of long-term growth goals. Adaptability helps them integrate these pragmatic needs with personal aspirations (Soares and Taveira, 2024; Zhao and Guo, 2010). Finally, students factor in *life pursuits*, such as work–life balance, geographic preference, and organizational culture, where control and concern dimensions of adaptability play a key role (Gyarteng-Mensah et al., 2022; Sortheix et al., 2015).

These four dimensions provide a concise lens on engineering students' career motivation, demonstrating how personal aspirations intersect with external constraints. The internal–external split is consistent with contemporary value-based accounts (Lipshits-Braziler et al., 2025) and with Chinese HE/employability evidence emphasizing labor-market cues, employer reputation and development pathways (Dai and Pham, 2024).

### 5.3 Cultural framing of career values

Compared with the status-oriented choices of the 1980s, which waned by the late 1990s (Ling et al., 1999), today's Chinese engineering students prize financial security and organizational stability, a pattern reinforced by intense market competition and family expectations in a collectivist culture (Zhang and Yang, 2024). While global findings show that graduates gravitate toward reputable employers with clear growth prospects (Purohit et al., 2021), the participants further highlight a strong emphasis on long-term preparation. Contrary to the leisure-focused orientations reported by Greenberger and Steinberg (1986), they use career adaptability to align ambition with cultural and economic realities.

### 5.4 Extending SCCT through value-informed adaptability

While this study primarily interprets the link between values and decision-making through the lens of career adaptability, the findings also extend the SCCT. SCCT highlights the role of self-efficacy, outcome expectations, and goals in shaping career paths (Lent et al., 1994), but has been critiqued for under-emphasizing the role of career values as motivational foundations (Hirschi, 2010; Wang, 2021). The results help fill this gap by showing how well-articulated career values, such as autonomy, competence, and growth, motivate and sustain proactive behaviors. These values, channeled through the adaptive resources of concern, control, curiosity, and confidence, shape concrete choices under uncertainty. This study thus enriches SCCT by clarifying how value-driven adaptability bridges internal motivation and goal-directed action, especially in high-pressure, collectivist contexts like contemporary China.

## 6 Conclusion and implications

This study examines how Chinese engineering students convert career values into career choices, mediated by career adaptability. Qualitative evidence from eleven participants reveals that well-defined values enhance students' career adaptability. When filtered through the four adaptive resources (concern, control, curiosity, and confidence), these values inform strategic career decisions that balance personal ambition with the realities of the labor market.

Two value dimensions emerge. *Intrinsic career readiness* captures pragmatic, independent, knowledge-centered and competence-building orientations that tackle early-career uncertainty. *Extrinsic career aspirations* cover leadership, challenges transforming into motivators, recognition, and satisfying workplace environments, signaling a desire to integrate personal growth with social contribution, far broader than the status-seeking values documented in the 1990s.

Career choice considerations likewise cluster into two domains. *Value embodiment* (personal growth, identity construction) translates readiness and aspirations into concrete developmental goals. *Material needs* (work compensation, life pursuit) anchor those goals in financial security, location, and organizational culture. Students continually balance these domains in a holistic calculus of meaning vs. manageability.

Career adaptability mediates these links. Concern prompts long-range scenario planning; control channels perseverance into staged skill acquisition; curiosity widens the information net; confidence converts mastery values into stretch assignments. Collectively the four “C”s form a renewable psychological resource that helps students reconcile fast-moving market pressures with their “new engineering” mandate to serve national innovation.

Practical implications follow. First, value-clarification workshops should precede skills-training: students who articulate what they care about build adaptability faster and make cleaner choices. Second, career-services units can foster adaptability directly through structured internships, reflective coaching and challenge-based projects that exercise the four “C”s. Third, employers seeking agile engineering talent should spotlight learning pathways, transparent promotion ladders, and supportive climates, as these elements simultaneously fulfill students' value commitments and material expectations.

Policy implications are equally clear. China's push for “new engineering” talent requires graduates able to pivot across emerging technologies; cultivating adaptability at university level is thus a workforce priority. Funding schemes that couple industry placements with academic credit can strengthen the concern–curiosity cycle, while scholarships tied to leadership or innovation roles can boost confidence and control.

In sum, clearly values give direction; adaptability supplies motion. Engineering students who align both are better positioned to navigate uncertainty, meet societal expectations, and craft sustainable careers. Future guidance programs should therefore target the value–adaptability nexus, ensuring China's next generation of engineers is both purpose-driven and market-ready.

## 7 Research limitations and future prospects

This study is subject to several limitations. First, the data derive from eleven in-depth interviews without triangulation, raising concerns about subjectivity and limiting the generalizability. Second, socio-economic indicators of interviewees were not collected; family income, parental education, or regional disparity may moderate how values and adaptability develop. Third, this study is purely qualitative; as such, cross-cultural claims are illustrative rather than statistically generalisable.

Future studies can address these gaps in four ways. (1) Mixed-method design. Combining surveys, focus group, and longitudinal follow-ups will bolster validity and reveal how values and adaptability evolve over time. (2) More diverse samples. Recruiting participants from multiple universities and regions, as well as international cohorts, would clarify which findings are culture-specific and which are universal. (3) Socio-economic lenses. Integrating variables such as family income, parental education levels may uncover how background resources shape the values–adaptability–career choices chain. (4) Quantitative model testing. A structural-equation or multilevel framework could test a conceptual model that links environmental factors (e.g., access to career resources) and psychological adaptations, thus mapping indirect and interaction effects more precisely.

Addressing these issues will deepen understanding of the pathways through which engineering students convert career values into choices and will guide educators and policymakers in tailoring interventions to diverse learner profiles.

## Data Availability

The raw data supporting the conclusions of this article will be made available by the authors, without undue reservation.
