# Supplementary material for: From values to choices: the mediating role of career adaptability of university engineering students
Source: Front Psychol. 2025 Dec 16;16:1712488. doi: 10.3389/fpsyg.2025.1712488 (PMC12748240; doi:10.3389/fpsyg.2025.1712488)
Supplement: Supplementary file 1 [file Supplementary_file_1.docx]

Supplementary Material

# Supplementary Data

**Section-A**

Supporting excerpts for career values (corresponding to Section 4.1 of the manuscript)

Excerpt A1 (Respondent B)

“If I enter a well-known company, even if I am doing the bottom job, I will still do it. I will learn about the development of the company through consulting with people around me, and if there is really development in this, I will choose and learn.”

Excerpt A2 (Respondent C)

“You cannot understand what it is like to start from the bottom (entry-level) unless you’ve been there. Join a student society, keep putting in the effort, and you’ll succeed.”

Excerpt A3 (Respondent H)

“There are times when I actually slack off, but I just have to push myself harder.”

Excerpt A4 (Respondent E)

“If you want to stand out, you must have certain domain-specific abilities to make yourself stand out, this time you can go to enhance your own aspects of the effort, for example, your ability to do things better than others, so that the leader will give you something to feel at ease, think you this person can, worth using a person, he will have to mention you that mind. Of course EQ is important in this environment, so that you can use your strengths and abilities more rationally to achieve certain successes for yourself.”

Excerpt A5 (Respondent G)

“If you give the same job to different people, the result will be different. That’s why I think it’s necessary to improve your ability to work and solve problems. You must be able to find the entry point quickly. This is something that you can only get experience of in practice, as you slowly work more and practice more. Find a way that suits you, a way to deal with some of the competencies, so that when you encounter a problem, you can identify and implement a solution more efficiently.”

Excerpt A6 (Respondent D)

“For university students, there is little point in going to university if they give up on themselves. To avoid getting lost it is still important to make yourself feel that learning is something very useful, such as enhancing professional knowledge through internship practice, like needing to design a circuit, laying out the actual thing, so that the profession can be enhanced by solving practical problems to really learn in.”

Excerpt A7 (Respondent H)

“The challenges allow me to consciously learn skills required by the company, thus preparing myself for real-world requirements. Through interacting with senior students from the department, I gained insights into the skills needed for specific professions and used this to prepare myself with relevant goals, such as becoming a hardware engineer.”

Excerpt A8 (Respondent E)

“When I first started tutoring, I was so nervous that I couldn’t even speak properly. Later, I became more confident when giving lessons and communicating with students.”

Excerpt A9 (Respondent F)

“I must learn how to study independently. The society is ever-changing. Even if I excel academically in school, I still need to constantly acquire new knowledge to keep up with industry trends. Without the ability to adapt and learn, I may fall behind.”

Excerpt A10 (Respondent J)

“When you set a dream and a goal, you encounter challenges, but solving them and moving forward is what fulfills you.”

Excerpt A11 (Respondent A)

“I really enjoy the white-collar lifestyle. Maybe it started from watching TV dramas when I was younger. That kind of look just feels great to me. I define ‘white-collar’ not necessarily as sitting in an office all day, since there are also white-collar jobs where people go out for business. But to me, it means having my own workspace, being able to complete tasks assigned by my boss, and being busy every day with lots of work to do. I love that feeling. There’s also the sense of routine, and having fixed work hours, meeting in a proper room. That makes me feel accomplished. I feel it’s a kind of life where I can rely on myself. I also associate white-collar jobs more with private companies. I have a good impression of private firms because the path for promotion is more transparent. If I do well, I get recognized by my leaders, and I think that’s important. In such jobs, I’d have more freedom to carry out tasks in my own way, shape my own little world at work, and stay busy and productive every day. That really appeals to me.

Excerpt A12 (Respondent C)

“First, the company should be large in scale. Also, it should offer enough opportunities for advancement and improvement. Overall, I want a workplace where I can feel I’m making progress, improving my skills, and gaining chances to learn and grow.”

Excerpt A13 (Respondent E)

“What’s more important is that the company is either growing, willing to grow, or has a strong reputation. Ideally, it’s a well-known name in the industry.”

Excerpt A14 (Respondent D)

“Although state-owned enterprises do not pay high salaries, they offer stable income and long-term recognition for your contributions. They also tend to be more human-centred.”

**Section-B**

Supporting excerpts and interpretations for career choice considerations (corresponding to Section 4.2 of the manuscript)

### Category 1: Value embodiment (Corresponding to Section 4.2.1 in the manuscript)

## 4.2.1.1 Theme 1: Personal growth opportunities

## *4.2.1.1.1 Sub-theme 1: Professional skill development and growth*

Several participants prioritized early, practical immersion as the key pathway to developing professional skills, long before formally entering their chosen fields. For them, growth was not an abstract goal but a deliberate, hands-on process rooted in authentic work experiences.

“My grades were solid and I even had scholarships, yet I poured most of my time into private tutoring. I wanted to step into the teaching field ahead of time and polish my professional abilities.” (Respondent F, Excerpt B1)

Here, F made a strategic choice: using tutoring not for income but as a training ground to rehearse real teaching responsibilities and refine classroom presence. This shows how skill development was not left to chance, but actively initiated to build readiness.

Likewise, H selected an internship not for its title or convenience, but for its potential to challenge technical capacity from day one:

“I took this internship precisely because it would throw real technical problems at me, nothing like school. From day one I was tackling issues all the way down to the basics, so I made myself learn the very professional skills this company relies on.” (Respondent H, Excerpt B2)

This example highlights a key mindset: seeking environments that simulate the pressures and expectations of real jobs, where learning is rapid, targeted, and necessary for performance. Such environments accelerate not just skill acquisition but also confidence and professional identity formation.

Together, these excerpts reveal a shared consideration: professional skill development requires early, intentional exposure to authentic challenges, and that the sooner one starts building those capabilities, the stronger the foundation for future success.

*4.2.1.1.2 Sub-theme 2: Supportive & equitable growth environments*

Participants’ career decisions were shaped not just by job content, but by whether the work environment would support fair and sustainable growth. They actively sought organisations where effort was recognized, development was supported, and progression depended on merit, not connections.

“In some family-run firms a relative gets the top slot, so no matter how hard you work you’ll never reach the position you want. Say I aim to be GM but the seat is reserved for a cousin. I’d rather join a company with clear rules where good performance actually moves you up; that feels truly fair.” (Respondent A, Excerpt B3)

This account highlights how the lack of mobility in closed systems deters long-term commitment, while transparent, rule-based structures are seen as worth investing in. Such clarity becomes a decisive factor when selecting employers, ensuring that career effort can lead to tangible rewards.

Beyond fairness, emotional support from both leaders and peers also influenced participants’ job decisions. Respondent J chose a role in part because positive feedback and a strong sense of belonging gave her the confidence to grow:

“I chose that job because the boss liked the way I worked and kept praising me. It really boosted my confidence. Back at college, a bunch of classmates would prep together; whenever there was a job fair we’d all pile onto the bus as one big group.” (Respondent J, Excerpt B4)

Here, the appeal of the position was not abstract. It stemmed from a concrete culture of encouragement and peer support, which made the workplace feel psychologically safe and energizing.

For others, the presence of long-term, hands-on mentoring played a key role. Respondent K described how continued support from an early boss shaped both career stability and entrepreneurial confidence:

“My very first boss has been helping me ever since… He said, ‘I’ll set up an office for you; come start your venture here, and no rent.’ Whenever a problem pops up he steps in, tells me what to say, and walks me through each stage of the start-up.” (Respondent K, Excerpt B5)

Such leadership not only reduced entry barriers but also turned the job into a platform for personal enterprise. It makes this environment particularly attractive for someone with start-up ambitions.

Collectively, these narratives show that supportive and equitable growth conditions are more than “nice-to-haves”. They are active filters in participants’ career decision-making. For early-career professionals, selecting a workplace with transparent rules, emotional reinforcement, and long-term developmental support directly shapes both short-term motivation and long-term career direction.

*4.2.1.1.3 Sub-theme 3: Career development resources*

When selecting early-career jobs, participants didn’t just focus on income or prestige. They weighed whether a role could unlock future growth. This meant paying attention to learning opportunities, qualification pathways, and promotion structures embedded in the organisation or profession.

Respondent B, for instance, was willing to accept a lower starting point in exchange for joining a reputable, upwardly mobile company:

“If I can get into a well-known company, even in the lowest job, I’ll stick with it. I ask colleagues about the firm’s direction; as long as the business is truly growing and respected, that’s what I choose to learn from. Things like location, pay or title matter less to me than joining an organisation that’s expanding and well-regarded.” (Respondent B, Excerpt B6)

Internal learning potential and company trajectory outweighed short-term benefits. This participant deliberately chose settings where knowledge transfer and mobility were realistic, seeing that as a more strategic investment in the long run.

Respondent D took a different route, choosing a local teaching job because it allowed him to keep preparing for graduate school, and he viewed this as a credential essential to future advancement in education:

“In teaching, a higher degree really matters. I ultimately chose a high-school job close to home. Working nearby gives me the control and time I need to keep preparing for the next exam round.” (Respondent D, Excerpt B7)

Rather than opting for immediate ambition or status, D prioritized a job that offered temporal and logistical flexibility, making it possible to keep investing in career qualifications.

Meanwhile, a participant valued the civil aviation industry for its predictable and structured promotion ladder, a system that clearly rewards professional mastery:

“When I first looked into civil aviation, what struck me was the clear path: start as a pilot, build your hours and skills, and move up to captain.” (Respondent E, Excerpt B8)

The attraction here lies in the visible long-term return on training, where each level of skill is directly tied to higher income and rank. It’s a resource-rich environment for those willing to follow a disciplined trajectory.

Taken together, these examples show that young professionals actively scan for developmental infrastructure when making career choices. Whether through companies with momentum, roles that leave room for study, or industries with structured pipelines, what matters is access to tools that allow growth to unfold over time.

*4.2.1.1.4 Sub-theme 4: Alignment with personal attributes and work ethics*

Participants’ career decisions were often guided by self-knowledge, a realistic appraisal of their working temperament, personal preferences, and long-term sustainability. Rather than defaulting to prestige or external expectations, they selected career paths that aligned with how they function best.

“A job has to fit who you really are. Everyone dreams of the ‘best’ option, but the best isn’t always the right one… After a while I have to get up and move, so a purely academic master’s path just isn’t for me.” (Respondent B, Excerpt B9)

The decision to forgo a research-intensive path was not due to a lack of ambition, but a strategic choice informed by deep awareness of one’s own limits and energy rhythms. This kind of self-assessment helped participants filter out incompatible options early.

Others factored in risk tolerance and lifestyle compatibility. Respondent A described how her conservative disposition and gendered perceptions of job security shaped her choice:

“Because of my personality and family background I’m fairly conservative, so a public (state) school feels steadier and suits a woman better. I work late almost every night. Anything to be sure I’ll never look back and regret my choice.” (Respondent A, Excerpt B10)

In this case, the public-sector role wasn’t chosen because of its symbolic value, but because it matched the participant’s preference for predictability and her long-term commitment ethic.

Entrepreneurial routes were also filtered through this self-alignment lens. Respondent K described how both personality and a strong sense of responsibility made entrepreneurship a fitting direction:

“I’m a restless self-starter by nature, so entrepreneurship is the career that truly matches both my sense of duty and my get-things-done temperament.” (Respondent K, Excerpt B11)

Entrepreneurship here isn’t romanticized. It is a pragmatic fit that allows this individual to operate in line with their natural working style and ethical standards.

Taken together, these cases demonstrate that career alignment is about fit rather than idealism. Participants deliberately chose roles that suit how they think, act and persist, because sustainable motivation often stems from doing what feels both natural and meaningful on a daily basis.

*4.2.1.1.5 Sub-theme 5: Motivation and aspirations*

Participants frequently grounded their career choices in a desire to improve life circumstances, both for themselves and their families. These aspirations were not presented as lofty ideals or abstract visions; instead, they reflected practical and personal considerations shaped by individual experiences and responsibilities. For some, the primary motivation was to enhance their own quality of life:

“To be honest, you don’t need that many goals. If you stick to just one, I want a better life for myself, that’s enough… My parents didn’t agree, but to me it reflects a better quality of life.” (Respondent C, Excerpt B12)

In this case, the career decision was driven by a desire to make steady, tangible improvements to one’s daily life, even if it involved going against parental expectations or traditional norms. The emphasis was on personal fulfilment through material and emotional well-being.

Other participants highlighted a strong sense of responsibility towards family members:

“My family isn’t wealthy or influential. if I don’t work hard now, I won’t be able to support my parents when they grow old. I just want to try harder, so that they can live better in the future.” (Respondent B, Excerpt B13)

Here, the motivation to pursue a stable and upward-moving career path stemmed from the hope of securing long-term support for parents and creating better conditions for future generations. This sense of duty gave additional purpose to daily work efforts.

In other cases, participants were focused on breaking away from limiting environments:

“I grew up in a small county and want to change my circumstances. Young people shouldn’t settle… I look for work that gives me a bigger platform and a path upward. (Respondent G, Excerpt B14)

This statement reflected a strategic approach to job selection. Rather than accepting inherited constraints, G actively sought professional opportunities that promised visibility, development, and greater social mobility.

Taken together, these excerpts reveal that career aspirations were closely tied to lived experience. Participants chose jobs based not only on personal interest, but also on how effectively those jobs could help them improve life quality, honour family responsibilities, or pursue advancement beyond their backgrounds.

## 4.2.1.2 Theme 2: Identity construction

*4.2.1.2.1 Sub-theme 1: Professional respect*

For these engineering students, the career choice considerations were closely linked to the desire to earn genuine professional respect that comes from competence, credibility, and authentic contribution within their field. One recalled how the authority of high school teachers left a deep impression:

“At that time, I felt that high school teachers had a strong presence in the classroom. It carried real weight. Being acknowledged by others meant a lot.” (Respondent J, Excerpt B15)

This reflection highlights how professional presence and societal recognition helped shape J’s career decision to become a teacher. It was not just a job, but a role associated with influence and esteem in the eyes of students and the wider community.

A similar value emerged in the reflections of Respondent K, who spoke about remaining in the technical field despite the challenges of transitioning into sales roles:

“Switching from tech entrepreneurship to sales is hard, but a technical background coupled with business sense allows me to tackle every issue from an engineering angle. In this field, it’s hands-on achievements, not paper honors, that earn real professional respect.” (Respondent K, Excerpt B16)

The sense of respect was rooted in practical expertise and the ability to solve problems, rather than in formal qualifications or symbolic recognition. This outlook strongly influenced K’s decision to remain in a technical track, where the standards of respect were aligned with personal values and skills.

Together, these accounts illustrate that for some early-career professionals, respect is not merely an external reward. It is a meaningful internal benchmark, guiding which roles feel worthwhile and which work environments align with a deeper sense of occupational integrity.

*4.2.1.2.2 Sub-theme 2:* *Social recognition*

While some participants emphasized respect earned through professional expertise, others were more strongly motivated by the desire for broader social recognition. Rather than focusing on acknowledgment from within a profession or workplace, these individuals sought validation from family, peers, and society at large. The recognition they valued was less about technical competence and more about symbolic status, pride, and public admiration.

Respondent E reflected on how certain professions, such as piloting, carry inherent prestige in the eyes of others:

“Among university students, being a pilot is seen as a very noble profession. It’s considered quite a special career.” (Respondent E, Excerpt B17)

The perception of piloting as an honorable and elite path added to its appeal, not only because of its responsibilities but also because of how it was regarded by those outside the profession. Choosing such a career was, in part, about achieving visible social status.

Similarly, Respondent I described how family pride and recognition shaped career motivation:

“I hope to become a source of pride for my parents. I have an uncle who went to vocational school because he couldn’t afford high school. Even so, after he got a job, my grandparents felt really proud. Maybe because of his example, my mum always speaks about him with such pride.” (Respondent I, Excerpt B18)

This account highlights how inter-generational examples and familial expectations influenced occupational choices. Being admired by one’s family was a powerful driver, especially in comparison to previous generations. Career success was closely tied to fulfilling family hopes and being seen as a person of value and achievement.

Together, these excerpts demonstrate that for some young professionals, social recognition functions as a key factor in evaluating and selecting career paths. Unlike professional respect, which centers on internal standards of expertise, social recognition is rooted in how others, particularly family and peers, perceive one’s role and accomplishments.

*4.2.1.2.3 Sub-theme 3: Self-fulfilment*

Some participants made career decisions based on the desire for self-realization and alignment with personal ideals. For Respondent A, the appeal of a career lies not just in the role itself, but in how it reflects a preferred lifestyle and identity. A explained,

“My dream day is staying busy but on my own terms, carving out my own little world. You only live once, so pick what you love and have no regrets. I’m drawn to a white-collar life, wearing suits, being a ‘girl boss’, having coffee in meetings. That is the state in which I feel fulfilled.” (Respondent A, Excerpt B19)

This shows how professional preferences can be shaped by the pursuit of autonomy and self-expression, influencing the type of work environment and career pathway one finds most meaningful.

Others emphasized the importance of daily emotional rewards when deciding on long-term career paths:

“Each time a student asks me a question in class, I feel a sense of accomplishment. That sense of impact is why I’m determined to stay in teaching for the long haul.” (Respondent F, Excerpt B20)

In this case, the decision to remain in teaching stems from a strong emotional connection to the role and the affirmation that comes from helping others, which are factors that support long-term professional commitment.

The driving force behind career choice was also the opportunity to contribute to a larger social good:

“I’ve always had a strong desire to realize my personal value. I chose to study technical skills so I could serve the people.” (Respondent E, Excerpt B21)

Here, vocational training is not only a means of employment but a deliberate step toward meaningful contribution, showing how self-fulfillment and social responsibility can align in shaping professional direction.

*4.2.1.2.4 Sub-theme 4: Leadership recognition*

Recognition from supervisors played a meaningful role in shaping participants’ career decisions. For some, encouragement from leadership strengthened confidence and commitment to stay in a role. As Respondent J reflected,

“While I was working, my boss kept praising me, and that boosted my confidence enormously. That positive feedback from leadership reinforced my decision to stay in the role.” (Respondent J, Excerpt B22)

Here, the appreciation shown by a manager not only affirmed J’s sense of competence but also served as a deciding factor in continuing the job.

In other cases, career paths were influenced by long-standing support from leaders who offered more than praise. Respondent K shared:

“From the very start, the boss at my first company recognized my potential and, for ten straight years, has kept backing me, even when I launched my own venture. Because I always showed him respect, he acknowledged my value, offering office space, resources and guidance. He’s the key sponsor who believes that if he helps you, you’ll honour that trust.” (Respondent K, Excerpt B23)

This highlights how trusted leadership can shape long-term career decisions, with sustained mentorship and practical support creating opportunities for advancement and entrepreneurship. In such cases, the quality of leadership becomes a decisive factor in where and how participants choose to build their careers.

**Category 2: Material needs (Corresponding to Section 4.2.2 in the manuscript)**

**4.2.2.1 Theme 1: Work compensation**

*4.2.2.1.1 Sub-theme 1: Company size*

Company size was an important factor in shaping participants’ career preferences, often associated with perceptions of stability, opportunity, and long-term development. Some participants actively sought out large, well-established firms with strong reputations.

“I tend to prefer large firms. Ideally, I’d go for industry giants like BAT, Huawei, ZTE, or Datang. These are companies with real strength and serious name recognition.” (Respondent C, Excerpt B24)

The attraction to large companies stemmed from their visibility, infrastructure, and perceived capacity to offer structured career paths.

Others were influenced by tangible indicators of scale and growth potential. For example, Respondent K described how the company’s financial and operational scale helped inform his decision to stay:

“When I was in Shenzhen, the boss said their office rent alone was over two million a month, and each square meter cost 1.3 million. The company pulls in about 600 million in annual sales. Those figures showed me just how big the operation is; with resources that deep and a market that large, the growth potential was obvious, which is why I chose to stay on.” (Respondent K, Excerpt B25)

The scale of operations signaled long-term potential and stability, making the company a desirable place for career development. However, not all participants could enter large firms right away. Respondent I shared a more pragmatic experience:

“At first, I really wanted to land a job at a big company, that was the dream. But reality hit. As a total newcomer, I started off earning just 2,500 yuan a month, with rent to pay. So I told myself: stay grounded, take each step seriously, and whatever task you’re given, master it fully.” (Respondent I, Excerpt B26)

This example shows how early-stage job seekers often adjust their expectations when faced with financial pressures or limited options. For students like I, long-term goals remained tied to large firms, but initial choices were shaped by the need to gain experience and build credibility.

Together, these excerpts illustrate that company size, both in reputation and in scale, can be a powerful influence on career decision-making, though early experiences may also require flexibility and adaptation.

*4.2.2.1.2 Sub-theme 2: Company development prospects*

Participants often assessed a company’s future trajectory when making career decisions. Rather than focusing solely on salary or current position, they prioritized long-term potential and sought employers that showed signs of expansion, ambition, and a clear path forward. K expressed a strong belief in the company’s direction:

“I’m very confident about the company’s future. I hope that within ten years I can help take it public.” (Respondent K, Excerpt B27)

Here, the company’s anticipated growth is not only a reason to stay but a source of motivation, anchoring K’s long-term commitment in a shared future vision. For students like K, career development was closely tied to the organization’s larger ambition, making personal goals inseparable from collective progress.

Similarly, Respondent B described a pragmatic approach:

“What matters most is that the company is growing or willing to grow. If I can see room for development there, I’ll stick with it. I ask people around me to understand the company’s direction. If it really has potential, that’s what I’ll choose.” (Respondent B, Excerpt B28)

This response emphasized the importance of forward movement in both organizational and individual. Rather than taking jobs based on static status, it made decisions based on active inquiry into the company’s growth potential. Developmental space, not short-term incentives, drove long-term employment decisions.

Together, these accounts suggest that early-career professionals value companies with clear, promising trajectories. Career choices are shaped not just by what the company is today, but by what it signals it might become.

*4.2.2.1.3 Sub-theme 3: Salary*

For many participants, salary was not just a matter of personal gain but a practical factor in shaping career decisions. Rather than pursuing idealized roles, they often made choices based on whether a job could provide long-term financial stability and meet family obligations.

Respondent D emphasized the importance of evaluating return on investment:

“Financial independence is really the key. I keep asking myself whether the time and money I put in will pay off. I want to give my parents and my younger brother a stable life.” (Respondent D, Excerpt B29)

Here, salary functions as a benchmark for career value. D’s decision-making centers on whether the effort required by a role is balanced by sufficient income, especially when extended to supporting dependents. The goal is not only to sustain oneself, but also to ensure economic stability for family members.

Respondent E similarly viewed income potential as a central factor:

“When I first looked into civil aviation, I thought, if I could become a pilot, the pay and benefits would probably be good. My parents are getting older and can’t do hard labor anymore, we’ll be the ones supporting them.” (Respondent E, Excerpt B30)

In this case, the appeal of a high-paying career lies in its practicality. The earning potential of piloting was attractive not only for status or interest but for its clear role in fulfilling inter-generational financial responsibilities.

Together, these examples illustrate how salary considerations guide early career decisions. Rather than treating income as a bonus or symbolic marker, participants viewed it as a necessary condition for long-term commitment and familial responsibility.

*4.2.2.1.4 Sub-theme 4: Satisfying job description*

Several participants emphasized how the structure and content of the job itself influenced their career decisions. Whether seeking stability, comprehensive responsibility, or a clear evaluation system, they selected positions where the day-to-day tasks matched their expectations and work preferences.

For example, one described why a high school teaching position appealed:

“Teaching from Grade 10 through Grade 12 hardly introduces anything new; once you’re in that cycle you feel calm. You prep in advance and teach at most four periods a day, basically delivering the same lesson in different classrooms.” (Respondent J, Excerpt B31)

The predictable routine, moderate workload, and low variation created a sense of stability. For J, this regularity was not a drawback but a reason to commit to high school teaching, which is a role offering manageable effort and minimal surprises.

By contrast, Respondent K sought broad and ongoing responsibility:

“I wrote all the company’s software as a software engineer, and now I’m also handling product planning. I can take care of a lot by myself.” (Respondent K, Excerpt B32)

Here, the appeal lies in full-cycle involvement. The ability to influence everything from design to implementation gave K both ownership and autonomy—qualities that made the job engaging and worth continuing.

Respondent E selected a path defined by high standards and rigorous evaluation:

“To make captain you must first meet strict professional standards: your flying skills must be flawless and your sense of responsibility high. There’s an annual medical and additional exams at every stage, and fail any of them and you’re out.” (Respondent E, Excerpt B33)

For E, civil aviation’s structured promotion system, with transparent benchmarks and strict checks, was a major draw. The profession’s clarity and accountability created a strong sense of purpose and measurable progress.

These accounts show that job satisfaction is not only about passion or calling; it often comes down to how well the role’s actual tasks and demands align with a person’s work style, goals, and need for clarity or challenge.

**4.2.2.2 Theme 2: Life pursuit**

*4.2.2.2.1 Sub-theme 1: Favorable geographical location*

Geographical preferences played a key role in participants’ job decisions, often reflecting a strategic assessment of opportunities, cost of living, and long-term benefits. Rather than being guided by lifestyle ideals or emotional attachments, participants weighed the practical implications of location on career progression and personal sustainability.

Respondent D expressed a clear preference for economically dynamic regions:

“When job-hunting I also wanted a big city, so I planned on Shenyang. The gap between my home county and Shenyang is huge. Shenyang is now a ‘new first-tier’ city, while we’re just a small county.” (Respondent D, Excerpt B34)

For students like D, relocating to a higher-tier city like Shenyang was a deliberate choice to access broader job markets and stronger economic growth, using city status as a proxy for career potential.

“Back then I considered heading for first-tier places like Changchun, Beijing or Harbin, but in cities that ‘too good’ the income often isn’t worth what you have to put in. I’d rather pick a mid-level city and live a balanced life.” (Respondent F, Excerpt B35)

This view reflects a cost-benefit approach. Students opted for a mid-sized city where the workload and living conditions were better aligned with the income, indicating a rational evaluation of return on effort.

In contrast, Respondent H chose a high-pressure city based on long-term potential:

“I wanted to see a big city and broaden my horizons, convinced it offered more opportunities. So I went to Beijing in July, packed, sweltering subway and all, and stuck it out because the city itself was worth it.” (Respondent H, Excerpt B36)

H selected Beijing as a strategic platform to access concentrated resources, career connections, and development opportunities, accepting short-term discomfort in exchange for long-term professional gains.

Together, these decisions reflect how early-career professionals evaluate geography through the lens of advancement, weighing access to opportunity, financial return, and life management to guide their employment choices.

*4.2.2.2.2 Sub-theme 2: Organizational reputation*

Participants considered institutional reputation as a strategic factor in their employment decisions, focusing on how perceived quality and selectivity could affect career development and long-term standing. Rather than being drawn to symbolic prestige alone, they evaluated reputation through the lens of practicality and accessibility.

Respondent G explained:

“I was aiming for a top middle or high school. But thinking it over, if I had to choose between location and the school itself, I’d pick the school. Even if it’s in a less prominent place, I’d go for one that’s really strong locally. At our school, the staff in general are highly capable.” (Respondent G, Excerpt B37)

This account reflects a preference for strong institutional reputation at the local level. G prioritized a work setting with high professional standards and team competence, even if located outside major urban centers. The emphasis was on organizational substance rather than external prestige.

Similarly, Respondent H noted:

“I looked for a relatively good private school in Shenyang. When it comes to job-hunting, the gap between second-tier and top-tier university grads is most obvious. You often need to cross a very high threshold. Some schools only take free-teacher-training programme grads from elite institutions.” (Respondent H, Excerpt B38)

H’s account highlights how elite hiring criteria can limit access to well-regarded positions. Instead of targeting the most prestigious schools with high entry barriers, H strategically identified reputable but more accessible institutions, allowing for both status and feasibility in the career choice.

Overall, participants treated organizational reputation as a dynamic factor, valued not as a status symbol, but as a practical gateway to professional growth and workplace legitimacy within reach.

*4.2.2.2.3 Sub-theme 3: Working environment*

Participants actively considered the daily work environment when making career decisions, recognizing that the quality of interpersonal interactions and the broader atmosphere can significantly affect job satisfaction and sustainability. Rather than focusing solely on task content or salary, they weighed how team dynamics and workplace rhythm would influence their long-term commitment.

Respondent I described a deliberate strategy to assess the team environment:

“During my internship, when I joined the company I spent the first three months just watching. I’d bring my questions to the easy-going colleagues and keep my distance from the prickly ones. Only after I saw that the overall team spirit was friendly and supportive did I decide to stay.” (Respondent I, Excerpt B39)

This account highlights the importance of early-stage observation and selective engagement. I focused on identifying approachable colleagues and gauging overall team morale before choosing to stay, suggesting that the perceived social climate plays a critical role in job retention decisions.

Respondent J emphasized a different but related dimension:

“The work environment here is calm. Everyone’s sort of observing each other quietly. I looked around at the surroundings too. But once the workday ends, your time is your own. It all just feels pretty relaxed.” (Respondent J, Excerpt B40)

J valued a low-pressure environment where work does not spill into personal time, describing a setting that supports individual autonomy and mental clarity. The relaxed rhythm and clear work-life boundaries made the position more appealing.

Together, these accounts demonstrate that a positive work environment, whether defined by approachable peers, clear boundaries, or overall calm, can strongly influence whether individuals choose to join or remain in a role.

*4.2.2.2.4 Sub-theme 4: Distance from home*

Geographic proximity to home emerged as a key consideration in participants’ job decisions. Rather than pursuing positions in larger or more competitive cities, some individuals chose roles that allowed them to remain physically close to their families or property, reflecting a pragmatic approach to location-based constraints. As Respondent A explained:

“My current school in Panshi is fairly close to home, yet I still feel guilty for not being right beside my parents. That strong pull toward home is why I’ve turned down riskier jobs in farther-off cities.” (Respondent A, Excerpt B41)

This statement reflects how location decisions are shaped not only by convenience but by the perceived responsibility to stay within a manageable distance of family. The participant rejected potentially higher-return positions in more distant areas to maintain closer ties.

Respondent I shared a similarly practical rationale:

“I could move, but I won’t choose a city that’s too far from the house I bought, in a way I’ve set that limit for myself.” (Respondent I, Excerpt B42)

Here, property ownership serves as a fixed reference point that directly influences career mobility. The decision to remain within commuting range of a home investment reflects long-term planning and the need for locational stability.

These responses illustrate how proximity to home can define the geographic boundaries within which individuals explore and accept employment. For many, it serves as a decisive filter in selecting both job offers and career trajectories.

*4.2.2.2.5 Sub-theme 5: Organizational cultural*

Workplace culture played a notable role in shaping participants’ employment decisions. Rather than focusing solely on compensation or advancement, some individuals placed significant weight on whether the organizational environment encouraged open communication, mutual respect, and knowledge sharing.

One participant described how specific cultural features influenced long-term commitment:

“The first thing I value is gratitude, respecting mentors, parents and managers; the second is responsibility. I also enjoy open debate: when people voice opposing ideas, it sharpens everyone’s thinking. I stay where this ethos of respect, duty and candid discussion is the norm.” (Respondent K, Excerpt B43)

K’s comments reflect how structured, respectful dialogue and clear interpersonal norms can function as a selection criterion. When organizational culture supports open exchange and accountability, it becomes a reason to remain.

Similarly, Respondent I noted:

“When I joined, I asked questions freely and senior colleagues were happy to coach me. That supportive, sharing atmosphere convinced me to stay with the firm.” (Respondent I, Excerpt B44)

In this case, the availability of collegial support and accessible mentorship shaped the participant’s sense of fit. These elements signaled an environment conducive to learning and collaboration, which helped anchor the participant’s employment decision.

Together, these accounts suggest that job seekers evaluate not only the role itself but also the surrounding cultural dynamics. Environments marked by openness and support can encourage longer-term investment in a position.

**Section-C**

Supporting excerpts for career value–career choice considerations linkage through adaptability (corresponding to Section 5.1 of the manuscript)

Excerpt C1 (Respondent D)

“I don’t like studying, and my grade is not ideal, so instead of forcing myself to pursue graduate study, I plan to use these three years to engage with society, gain practical experience, and build a foundation. Compared to graduate students, my starting point may be lower, but while they spend three years studying in graduate schools, I will spend three years working. After those years, I can’t say for sure who will have a higher salary.”

Excerpt C2 (Respondent A)

“I don’t want a stable life. My teacher said girls should seek stability, but I’ve always dreamed of joining the private sector because it has been my goal since childhood.”

Excerpt C3 (Respondent D)

“My career plan is to enter senior management, where I will meet inspiring people in a better environment. Interacting with these people will motivate me to enhance my skills and broaden my vision.”

Excerpt C4 (Respondent E)

“I feel confident in my ability to start from the bottom, By taking one step at a time, I can progress steadily toward my goals.”

Excerpt C5 (Respondent H)

“I think the first thing is to collect relevant information about the development of my major, such as the employment situation of my schoolmates and seniors, so that I can quickly understand the employment prospect of my major and use it as a reference basis for my career choice.”

Excerpt C6 (Respondent I)

“When it comes to the employment season, I ask seniors who have gone through job interviews to find out current interview criteria and employer requirements and to get relevant information for my career choice.”

Excerpt C7 (Respondent C)

“I believe that with excellent and solid professional knowledge as a foundation, I have the opportunity to enter top enterprises. At the same time, I will consistently strive to improve, love learning professional knowledge, aim for top-tier achievement, master cutting-edge technology, and continuously improve my professional abilities.
